# C-reactive protein concentrations, clinical presentation, and short-term outcome in dogs with acute vomiting: a retrospective primary care cohort

**DOI:** 10.3389/fvets.2026.1990737

**Published:** 2026-10-23

**Authors:** Sin-Wook Park, Seong-Hyeon Baek, Min-kyu Oh, Yoon-Jung Do, Woong-Bin Ro, Chang-Min Lee

**Affiliations:** 1 SKY Animal Medical Center, Gwangju, Republic of Korea; 2 ING Animal Medical Center, Busan, Republic of Korea; 3 Division of Animal Diseases & Health, National Institute of Animal Science, Rural Development Administration, Wanju-gun, Republic of Korea; 4 Department of Veterinary Internal Medicine, College of Veterinary Medicine, BK21 Four Project Team and Animal Medical Institute, Chonnam National University, Gwangju, Republic of Korea

**Keywords:** acute phase protein, emesis, gastrointestinal signs, inflammation, prognosis

## Abstract

**Background:**

Vomiting is a common reason for dogs to be presented to veterinary hospitals and may accompany disorders of widely varying severity. C-reactive protein (CRP) is a major acute-phase protein in dogs, but its clinical relevance in dogs with acute vomiting in primary care remains poorly defined. This study aimed to characterize initial plasma CRP concentrations, evaluate their associations with clinical presentation and short-term outcome, and describe their distribution across underlying diagnoses.

**Methods:**

Medical records of 481 client-owned dogs presenting to a primary care hospital with vomiting of less than 2 weeks duration and undergoing CRP measurement at initial presentation were retrospectively reviewed. Dogs were categorized as Group A (<10 mg/L), Group B (10 to <35 mg/L), or Group C (≥35 mg/L).

**Results:**

The median plasma CRP concentration was 14 mg/L (interquartile range [IQR], 6–57 mg/L). Dogs were classified into Group A (*n* = 204), Group B (*n* = 119), and Group C (*n* = 158). Vomiting frequency and the prevalences of reduced appetite and reduced activity differed significantly among the three groups (all *p* < 0.001), with higher values in Group C than in Groups A and B on pairwise comparisons. Definitive diagnoses were established in only 117 dogs (24.3%), with substantial overlap in CRP concentrations among underlying diseases. The 14-day mortality rate differed significantly among the groups (*p* < 0.001) and was higher in Group C than in Groups A and B (8.2% vs. 1.0 and 1.7%, respectively). Non-surviving dogs had significantly higher CRP concentrations than surviving dogs (*p* < 0.001). However, among 44 dogs with CRP concentrations >100 mg/L, only two (4.5%) died within 14 days.

**Conclusion:**

In this primary care cohort, higher initial CRP concentrations were associated with more frequent vomiting, reduced appetite, reduced activity, and increased 14-day all-cause mortality. Nevertheless, most dogs with CRP concentrations >100 mg/L survived the 14-day follow-up period. Thus, although CRP was associated with mortality at the cohort level, marked CRP elevation alone should not be equated with a grave short-term prognosis in an individual dog presenting with acute vomiting.

## Introduction

Vomiting is among the most common reasons for dogs to be presented to veterinary practices (1, 2). Clinical courses range from self-limiting episodes to severe disease requiring urgent diagnostic evaluation and treatment (3, 4).

Vomiting is a non-specific clinical sign that can occur in various diseases and is classified into gastrointestinal or non-gastrointestinal categories (5). Non-gastrointestinal disorders include diseases of the pancreas, liver, and kidneys, as well as endocrine, cardiovascular, or central nervous system diseases (6). The causes of gastrointestinal disorders can be infectious, oncogenic, mechanical, toxic, or non-infectious inflammatory (4, 6). Therefore, multiple differential diagnoses must be considered when assessing a dog with vomiting. In general, diagnosis is made through history taking, clinical signs, physical examination, laboratory testing, and diagnostic imaging procedures such as radiography and abdominal ultrasonography (5, 7).

C-reactive protein (CRP) is commonly used as a marker of inflammation in dogs (8, 9). Although it is non-specific, it is a highly sensitive marker that markedly increases in various diseases with inflammation (8–11). Additionally, CRP has been studied as a prognostic marker in gastrointestinal diseases such as parvovirus infection, pancreatitis, and acute abdomen (8, 9, 12). Previous canine studies have also used CRP thresholds to assess systemic inflammation, with concentrations >35 mg/L used to define systemic inflammation (13–15). However, its utility in dogs with acute vomiting in primary care settings remains poorly defined.

Despite its widespread clinical use, the clinical significance of CRP concentrations in dogs presenting with acute vomiting in primary care settings remains incompletely characterized. In these settings, a definitive cause often remains unidentified, and initial clinical decision-making relies heavily on the presenting clinical features and available diagnostic information. Thus, rather than focusing solely on the diagnostic value of CRP for specific etiologies, understanding how CRP relates to clinical presentation and short-term outcome may be of greater practical importance. This retrospective study aimed to characterize plasma CRP concentrations in dogs presenting with acute vomiting to a primary care hospital and to evaluate their associations with clinical presentation, underlying diagnoses, and short-term outcome.

## Materials and methods

Medical records were retrospectively reviewed for all dogs presenting with the chief complaint of vomiting to ING Animal Hospital, Busan, South Korea, between September 2021 and November 2024. Acute vomiting was defined as vomiting present for less than 2 weeks. Dogs were included if vomiting had been present for less than 2 weeks and CRP measurement had been performed at the first presentation, before any therapeutic intervention. Cases were excluded if the dogs had been treated for gastrointestinal diseases within 3 months prior to the first presentation or if follow-up data were unavailable.

Data were tabulated in an electronic spreadsheet (Microsoft Excel 365, Microsoft Corp., Redmond, WA, USA). The collected information included age, breed, sex, body weight, underlying medical conditions, physical examination findings, clinicopathologic data, and diagnostic imaging findings. Blood analysis was performed before initiation of therapy. The duration and frequency of vomiting and concurrent clinical signs (diarrhea, reduced appetite, and reduced activity) were obtained from the history. The duration of hospitalization, vital status at discharge, and treatments administered were also recorded. Follow-up information through day 14 was obtained from medical records, subsequent veterinary visits, and telephone consultations. The primary outcome was all-cause mortality within 14 days after the initial presentation.

Plasma CRP concentration was measured using a point-of-care analyzer (VetChroma; Boditech Med Inc., Republic of Korea) (16). The manufacturer’s reference interval was <10 mg/L, and plasma CRP concentrations ≥10 mg/L were considered elevated.^1^ The analytical measurement range was 5–500 mg/L. Values below 5 mg/L were recorded as 5 mg/L, and values above 500 mg/L were recorded as 500 mg/L.

Other diagnostic methods included hematology analysis,^2^ plasma biochemistry profile assessment,^3^ measurement of canine pancreas-specific lipase (cPL),^4^ urinalysis, abdominal radiography, ultrasonography, barium contrast study, parvovirus/coronavirus antigen testing,^5^ rectal scrapings, and an adrenocorticotrophic hormone (ACTH) stimulation test (5 μg/kg IV).^6^ In cases where additional diagnostic procedures or intensive care were required, dogs were referred, and the relevant data were obtained.

Based on the initial plasma CRP concentrations, dogs were stratified into three mutually exclusive groups: Group A, <10 mg/L; Group B, 10 to <35 mg/L; and Group C, ≥35 mg/L. The 10 mg/L boundary was based on the manufacturer’s reference interval (<10 mg/L). The 35 mg/L boundary was selected based on previous canine studies that used CRP concentrations ≥35 mg/L to define systemic inflammation (13–15).

All statistical analyses were performed using commercial software (IBM SPSS Statistics, version 27, IBM Corp., Armonk, NY, USA). The normality of continuous variables was assessed using the Shapiro–Wilk test. Continuous variables are reported as the mean ± standard deviation (SD) or median (interquartile range, IQR), as appropriate, and categorical variables as numbers and percentages. The chi-square test was used to compare the distributions of clinical signs and the 14-day mortality rate among Groups A, B, and C. When a significant overall difference was identified, pairwise comparisons between groups were performed with adjustment for multiple comparisons using the Holm method. Fisher’s exact test was used instead of the chi-square test when expected cell counts were small. Plasma CRP concentrations were compared between surviving and non-surviving dogs using the Mann–Whitney U test. The 95% confidence intervals (CI) for median CRP concentrations in surviving and non-surviving dogs were estimated using the percentile bootstrap method. Statistical significance was defined as *p* < 0.05.

## Results

### Characteristics

The initial search of medical records identified 943 dogs presenting with acute vomiting. Of these, 462 were excluded because no diagnostic testing was performed (*n* = 338), follow-up information was unavailable (*n* = 68), or diagnostic testing was performed without CRP measurement (*n* = 56), leaving 481 dogs for inclusion in the study. The dogs represented 24 different breeds, with Miniature Poodles being the most frequently represented breed (*n* = 98, 20.4%), followed by Pomeranians (*n* = 73, 15.2%), Maltese (*n* = 72, 15.0%), mixed-breed dogs (*n* = 71, 14.8%), Bichon Frises (*n* = 59, 12.3%), Chihuahuas (*n* = 17, 3.5%), Yorkshire Terriers (*n* = 15, 3.1%), and Shih Tzus (*n* = 14, 2.9%). The median age at presentation was 4.5 years (IQR, 2.0–8.3 years). Of the 481 dogs, 263 (54.7%) were male, of which 244 (92.8%) were neutered, and 218 (45.3%) were female, of which 178 (81.7%) were spayed. The median body weight was 4.5 kg (IQR, 3.3–6.4 kg).

Twenty dogs did not undergo any additional diagnostic tests. Abdominal radiography was the most frequently performed additional test (*n* = 326, 67.8%), followed by the cPL test (*n* = 289, 60.1%), CBC (*n* = 248, 51.6%), abdominal ultrasonography (*n* = 225, 46.8%), blood chemistry analysis (*n* = 166, 34.5%), rectal scraping tests (*n* = 80, 16.6%), and the ACTH stimulation test (*n* = 8, 1.7%). A total of 73 dogs tested positive on the SNAP cPL test. An underlying diagnosis was established in 117 of 481 dogs (24.3%) (Table 1). Cases in which no definitive diagnosis was established from the diagnostic tests performed were classified as having no definitive diagnosis. The most frequently diagnosed conditions were pancreatitis (*n* = 45, 9.4%), gastric foreign body (*n* = 21, 4.4%), and small intestinal foreign body (*n* = 12, 2.5%).

**Table 1:** Diagnosis of 481 dogs with vomiting.

Diagnosis	Total n	CRP, median (IQR), mg/L	Group A (*n* = 204)	Group B (*n* = 119)	Group C (*n* = 158)
			*n* (%)	*n* (%)	*n* (%)
No definitive diagnosis	364	10.0 (5.0–35.3)	182 (89.2%)	90 (75.6%)	92 (58.2%)
Pancreatitis	45	51.0 (20.0–96.0)	5 (2.5%)	11 (9.2%)	29 (18.4%)
Gastric foreign body	21	9.0 (6.6–28.8)	12 (5.9%)	6 (5.0%)	3 (1.9%)
Small intestinal foreign body	12	41.0 (21.7–116.5)	1 (0.5%)	4 (3.4%)	7 (4.4%)
Pyometra	6	106.2 (92.8–146.3)	0 (0%)	0 (0%)	6 (3.8%)
Abdominal mass	6	81.0 (48.8–91.3)	0 (0%)	1 (0.8%)	5 (3.2%)
Kidney disease	6	22.4 (12.4–59.3)	1 (0.5%)	3 (2.5%)	2 (1.3%)
Intoxication	5	14.2 (6.4–57.0)	2 (1.0%)	1 (0.8%)	2 (1.3%)
Acute hemorrhagic diarrhea syndrome	4	87.0 (74.3–178.9)	0 (0%)	0 (0%)	4 (2.5%)
Coronavirus infection	2	151.0 (79.0–223.0)	1 (0.5%)	0 (0%)	1 (0.6%)
Chronic inflammatory enteropathy	2	38.5 (28.3–48.8)	0 (0%)	1 (0.8%)	1 (0.6%)
Gall bladder rupture	2	56.5 (35.3–77.8)	0 (0%)	1 (0.8%)	1 (0.6%)
Parvovirus infection	2	106.0 (92.5–119.5)	0 (0%)	0 (0%)	2 (1.3%)
Hepatic disease	1	27.1	0 (0%)	1(0.8%)	0 (0%)
Immune-mediated hemolytic anemia	1	177	0 (0%)	0 (0%)	1 (0.6%)
Immune-mediated thrombocytopenia	1	80	0 (0%)	0 (0%)	1 (0.6%)
Hypoadrenocorticism	1	58	0 (0%)	0 (0%)	1 (0.6%)

Percentages were calculated within each CRP group. For diagnoses represented by a single dog, the individual CRP concentration is reported without an IQR.

The most common concurrent diseases were myxomatous mitral valve disease (*n* = 16, 3.3%), followed by hyperadrenocorticism (*n* = 11, 2.3%), chronic kidney disease (International Renal Interest Society [IRIS] stages 1–2; *n* = 7, 1.5%), mammary gland tumors (World Health Organization stage I; *n* = 6, 1.2%), hypothyroidism (*n* = 3, 0.6%), hypoadrenocorticism (*n* = 2, 0.4%), and benign prostatic hyperplasia (*n* = 1, 0.2%).

### Group comparison

The median plasma CRP concentration was 14 mg/L (IQR, 6–57 mg/L). Based on plasma CRP concentrations, the 481 dogs were classified into three groups: Group A (*n* = 204), Group B (*n* = 119), and Group C (*n* = 158). Among all dogs, 44 (9.1%) had extremely high plasma CRP concentrations (>100 mg/L). The median duration of vomiting was 1 day (IQR, 0–2 days). Reduced appetite was reported in 285 dogs (59.3%), reduced activity in 221 dogs (45.9%), and diarrhea in 194 dogs (40.3%). Hematemesis was observed in 29 dogs, and hematochezia was observed in 30 dogs with diarrhea. The distribution of vomiting frequency and the prevalences of reduced appetite and reduced activity differed significantly among the three groups (all *p* < 0.001). Pairwise comparisons showed that Group C had more frequent vomiting and higher prevalences of reduced appetite and reduced activity than Groups A and B (all *p* < 0.05), whereas no significant differences were observed between Groups A and B. The prevalences of diarrhea, hematemesis, and hematochezia did not differ significantly among the three groups (Table 2).

**Table 2:** Clinical presentation and 14-day mortality according to plasma CRP levels in 481 dogs.

Variables	Group A (*n* = 204)	Group B (*n* = 119)	Group C (*n* = 158)	Overall *p*-value
	*n* (%)	*n* (%)	*n* (%)	
Clinical signs				
Vomiting frequency				< 0.001
1–2 times/day	107 (52.5%)^a^	49 (41.2%)^a^	37 (23.4%)^b^	
3–7 times/day	75 (36.8%)^a^	56 (47.1%)^ab^	82 (51.9%)^b^	
8 or more times/day	22 (10.8%)^a^	14 (11.8%)^a^	39 (24.7%)^b^	
Hematemesis	15 (7.4%)	10 (8.4%)	4 (2.5%)	0.073
Diarrhea	73 (35.8%)	52 (43.7%)	69 (43.7%)	0.218
Hematochezia	10 (4.9%)	8 (6.7%)	12 (7.6%)	0.558
Reduced appetite	104 (51.0%)^a^	58 (48.7%)^a^	123 (77.8%)^b^	< 0.001
Reduced activity	61 (29.9%)^a^	46 (38.7%)^a^	114 (72.2%)^b^	< 0.001
Clinical outcome				
Mortality	2 (1.0%)^a^	2 (1.7%)^a^	13 (8.2%)^b^	< 0.001

Different superscript letters (a, b) within the same row indicate significant differences between groups based on Holm-adjusted pairwise comparisons (*p* < 0.05).

Median plasma CRP concentrations varied considerably across underlying diseases. Descriptively higher median CRP concentrations were observed in dogs with conditions such as pyometra, acute hemorrhagic diarrhea syndrome, and pancreatitis; however, substantial overlap was present across etiologies, including cases with no definitive diagnosis (Table 1).

### Treatment

The majority of dogs received standard supportive medical management, most commonly including gastroprotectants and antiemetics. Specific interventions were performed based on the identified underlying diagnoses. Six dogs with gastric foreign bodies underwent endoscopic removal (*n* = 5) or surgical removal (*n* = 1). Nine of 12 dogs with small intestinal foreign bodies underwent surgical removal of the foreign body. All dogs with pyometra (*n* = 6) underwent ovariohysterectomy. Three dogs with abdominal masses underwent surgical mass removal and were subsequently diagnosed with splenic hemangiosarcoma (*n* = 2) or an abdominal mast cell tumor (*n* = 1).

### Prognosis

A total of 17 dogs died within 14 days after the first presentation. The 14-day mortality rate differed significantly among the three CRP groups (overall *p* < 0.001). Group C had a significantly higher mortality rate (13/158, 8.2%) than Group A (2/204, 1.0%) and Group B (2/119, 1.7%) (both *p* < 0.05), whereas no significant difference was observed between Groups A and B. The median plasma CRP concentration in non-surviving dogs was 77 mg/L (IQR, 40.7–98.0 mg/L; 95% CI, 40.7–98.0 mg/L), which was significantly higher than that in surviving dogs (13 mg/L; IQR, 5.9–52.5 mg/L; 95% CI, 11.0–16.0 mg/L; *p* < 0.001) (Figure 1). Among the non-surviving dogs, kidney disease was the most common primary condition (*n* = 5), followed by pancreatitis (*n* = 3), abdominal mass (*n* = 2), and vomiting of unknown origin (*n* = 2). The remaining five dogs had parvovirus infection, gallbladder rupture, intoxication, pyometra, and acute hemorrhagic diarrhea syndrome (*n* = 1 each). Among the 44 dogs with extremely high plasma CRP concentrations (>100 mg/L), two (4.5%) died within 14 days. No definitive underlying diagnosis was established in 22 of these dogs (50.0%), all of which survived during the 14-day follow-up period.

**Figure 1:**
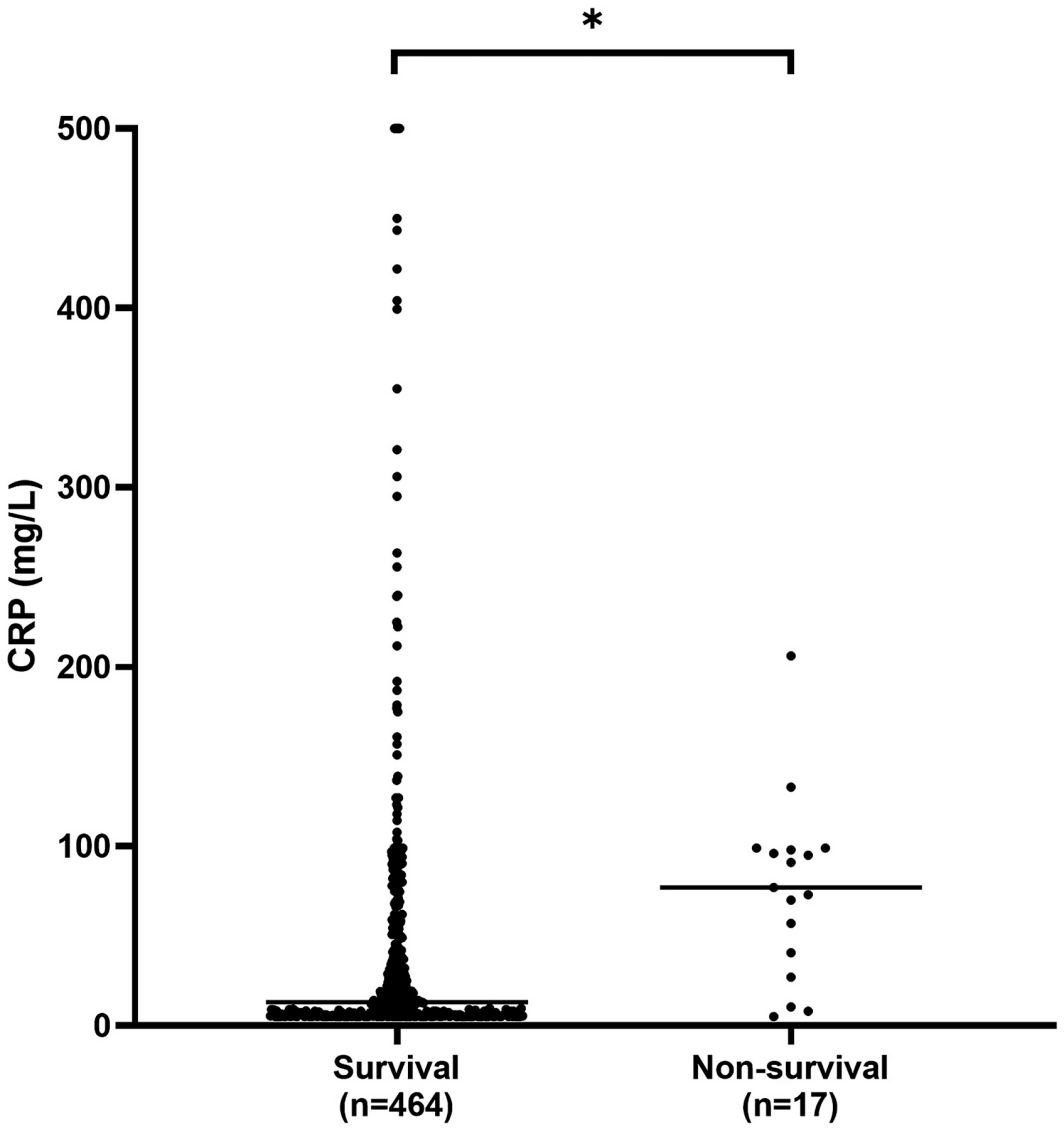
Plasma C-reactive protein (CRP) concentrations at initial presentation according to 14-day survival status. The median CRP concentration was 13 mg/L in surviving dogs and 77 mg/L in non-surviving dogs. Each dot represents an individual dog, and the horizontal line indicates the median. Plasma CRP concentrations were significantly higher in non-surviving dogs than in surviving dogs (*p* < 0.001).

## Discussion

In this study, we evaluated the clinical relevance of plasma CRP concentrations in dogs presenting with acute vomiting to a primary care hospital, focusing on associations with clinical presentation and short-term outcome and on descriptive distributions across underlying diagnoses. A definitive underlying diagnosis was established in only approximately one-quarter of dogs, limiting disease-specific interpretation of CRP concentrations. Despite this limitation, higher initial CRP concentrations were associated with more frequent vomiting and higher prevalences of reduced appetite and reduced activity and increased 14-day mortality. Importantly, although dogs with CRP concentrations ≥35 mg/L had higher 14-day mortality, extremely high CRP concentrations (>100 mg/L) did not consistently correspond to poor short-term outcomes. These findings suggest that, in this clinical setting, CRP may be more informative as an adjunctive marker of overall clinical status and short-term outcome than as an indicator of a specific underlying diagnosis.

In the present study, dogs in the highest CRP group had a significantly higher vomiting frequency and higher prevalences of reduced appetite and reduced activity, whereas the prevalences of diarrhea, hematemesis, and hematochezia did not differ significantly among the groups. This finding is consistent with previous assessments of disease severity in canine gastrointestinal disorders, in which variables such as attitude, appetite, and vomiting frequency have been incorporated into clinical evaluation (17–19). An association between CRP concentration and clinical disease activity has also been reported in dogs with inflammatory bowel disease (17). Taken together, these findings suggest that CRP may provide information on the overall clinical status of dogs with acute vomiting rather than reflecting any single gastrointestinal sign.

The association between CRP concentration and the underlying etiology was less clear. Although descriptively high CRP concentrations were observed in conditions such as pancreatitis, pyometra, acute hemorrhagic diarrhea syndrome, and abdominal masses, substantial overlap was evident across diagnoses. More than three-quarters of the dogs in this cohort did not receive a definitive diagnosis, reflecting the diagnostic challenges commonly encountered in dogs presenting with acute vomiting in primary care settings (3). These findings suggest that a single CRP measurement is more useful as an adjunctive marker of systemic inflammation than for identifying the specific cause of vomiting. CRP results should therefore be interpreted alongside the history, physical examination, and other diagnostic findings to guide further evaluation.

Furthermore, the substantial overlap in CRP concentrations among etiologies may partly reflect differences in the timing of blood sampling relative to disease onset. As a major acute-phase protein, CRP does not increase immediately after an inflammatory stimulus but rises over time as the systemic acute-phase response develops (8, 20). Consequently, dogs evaluated early in the disease course may have normal or only mildly increased CRP concentrations despite clinically relevant inflammation, including acute inflammatory conditions such as pancreatitis (21, 22). Accordingly, a normal or mildly increased CRP concentration at initial presentation should not be interpreted as definitive evidence of mild disease, particularly when the patient is evaluated early in the clinical course. This time-dependent response represents an inherent limitation of a single baseline CRP measurement; serial measurements may be more informative in selected patients, although this was not systematically evaluated in the present study (22–24).

The limitations of CRP as a standalone prognostic marker are further illustrated by the outcomes of dogs with extremely high CRP concentrations (>100 mg/L). Whereas a previous referral-hospital study reported a 3-month mortality rate of 37% among dogs with CRP concentrations >100 mg/L (25), only two of the 44 dogs (4.5%) with CRP concentrations >100 mg/L in the present study died within 14 days, and all 22 dogs without a definitive diagnosis survived. Although numerically different, these mortality rates are not directly comparable because of differences in patient selection, underlying disease distribution, and follow-up duration. Nevertheless, the survival of most dogs with extremely high CRP concentrations in the present cohort suggests that marked CRP elevation alone should not be equated with a poor short-term prognosis in an individual dog presenting with acute vomiting.

Several limitations of the present investigation must be acknowledged. First, the retrospective design limited the completeness and standardization of clinical data. Diagnostic work-ups were not standardized across dogs; consequently, some underlying diseases may have remained undetected, potentially contributing to the observed overlap in CRP concentrations among etiologies. Second, our prognostic evaluation was based solely on the initial CRP concentration. Although serial CRP measurements were obtained in some hospitalized dogs, the lack of standardized follow-up testing across the entire cohort precluded systematic evaluation of whether dynamic changes in CRP were associated with clinical outcomes. Third, because study inclusion required CRP measurement at initial presentation, selection bias cannot be excluded. Dogs selected for CRP testing may have differed systematically from those managed without CRP measurement, potentially according to perceived illness severity or diagnostic priorities. Fourth, the relatively high number of deaths among dogs with kidney disease warrants cautious interpretation because access to advanced supportive or referral-level interventions was limited in this primary care setting. Furthermore, owner-related factors, including financial limitations, may have influenced decisions regarding intensive treatment, referral, or euthanasia and thereby affected short-term outcomes independently of CRP concentration. Fifth, this was a single-center study that predominantly included small-breed dogs, potentially limiting the generalizability of the findings. The predominance of small-breed dogs likely reflects the regional companion dog population; however, the distribution of underlying diseases and the clinical significance of CRP may differ in populations with a greater proportion of large-breed dogs. Finally, the small number of deaths (*n* = 17) limited statistical power and precluded robust multivariable assessment of factors independently associated with short-term mortality. Collectively, these limitations preclude determining whether CRP concentration is independently associated with short-term mortality and do not permit causal inference regarding the relationship between CRP and clinical outcome.

In this primary care cohort of dogs with acute vomiting, higher initial CRP concentrations were associated with more frequent vomiting, reduced appetite, reduced activity, and increased 14-day all-cause mortality. Nevertheless, most dogs with extremely high CRP concentrations survived the 14-day follow-up period. These findings highlight the need to distinguish an association with mortality at the population level from a grave prognosis in an individual dog and to interpret CRP alongside the overall clinical assessment.

## Data Availability

The raw data supporting the conclusions of this article will be made available by the authors, without undue reservation.

## Footnotes

1 ANIVET Diagnostics Inc. Vet chroma™ cCRP instructions for use. Document No. INS-VC-CR, Rev. 05, March 5, 2018. Available at: https://weldonbiotech.com/wp-content/uploads/2018/05/ANIVET-INSERT_Vet_cCRP_20180305_Rev.05.pdf

2 Procyte One Analyzer, IDEXX Veterinary Diagnostics, United States.

3 Catalyst One Chemistry Analyzer, IDEXX Veterinary Diagnostics, United States.

4 SNAP cPL, IDEXX Veterinary Diagnostics, United States.

5 Canine parvovirus and canine coronavirus antigen test kits, Bionote Co., Republic of Korea.

6 ACTH preparation: Cynacten, Dalim BioTech, Republic of Korea. Cortisol assay: SNAP Cortisol, IDEXX Veterinary Diagnostics, United States.
